# NF2 signaling pathway plays a pro-apoptotic role in β-adrenergic receptor stimulated cardiac myocyte apoptosis

**DOI:** 10.1371/journal.pone.0196626

**Published:** 2018-04-30

**Authors:** Suman Dalal, Barbara Connelly, Mahipal Singh, Krishna Singh

**Affiliations:** 1 Department of Biomedical Sciences, James H Quillen College of Medicine, East Tennessee State University, Johnson City, TN, United States of America; 2 Center for Inflammation, Infectious Disease and Immunity, East Tennessee State University, Johnson City, TN, United States of America; 3 James H Quillen Veterans Affairs Medical Center, Mountain Home, TN, United States of America; Virginia Commonwealth University Medical Center, UNITED STATES

## Abstract

β-adrenergic receptor (β-AR) stimulation induces cardiac myocyte apoptosis *in vitro* and *in vivo*. Neurofibromin 2 (NF2) is a member of the ezrin/radixin/moesin (ERM) family of proteins. Post-translational modifications such as phosphorylation and sumoylation affect NF2 activity, subcellular localization and function. Here, we tested the hypothesis that β-AR stimulation induces post-translational modifications of NF2, and NF2 plays a pro-apoptotic role in β-AR-stimulated myocyte apoptosis.

**Methods and results:**

Treatment of adult rat ventricular myocytes (ARVMs) with β-AR agonist (isoproterenol) for 15 min increased phosphorylation (serine-518) and sumoylation of NF2. Co-immunoprecipitation assay confirmed β-AR-stimulated sumoylation of NF2. β-AR stimulation enhanced nuclear translocation of phosphorylated and sumoylated NF2. Specific inhibition of β_1_-AR and protein kinase A (PKA) decreased β-AR-stimulated increase in NF2 post-translational modifications, while inhibition of β_2_-AR had no effect. Activation of adenylyl cyclase using forskolin (FSK) mimicked the effects of β-AR stimulation. β-AR stimulation and expression of wild-type (WT)-NF2 using adenoviruses increased phosphorylation of mammalian sterile like kinase-1/2 (MST1/2) and yes activated protein (YAP), downstream targets of NF2. Knockdown of NF2 using siRNA in H9C2 cardiomyocytes decreased β-AR-stimulated increase in NF2 and YAP phosphorylation. siRNA-mediated knockdown of NF2 decreased β-AR-stimulated increase in apoptosis, while expression of WT-NF2 induced apoptosis in ARVMs. Expression of WT-NF2 stimulated the mitochondrial death pathway as evidenced by activation of c-Jun N-terminal Kinases (JNKs), and increase in cytosolic cytochrome c levels and Bax expression.

**Conclusion:**

β-AR stimulation affects post-translational modifications of NF2 via the involvement β_1_-AR/PKA/cAMP pathway, and NF2 plays a pro-apoptotic role in β-AR-stimulated myocyte apoptosis via the phosphorylation (inactivation) of YAP and involvement of mitochondrial death pathway.

## Introduction

Cardiac myocytes are generally considered as terminally differentiated cells, incapable of entering into the cell cycle [1,2]. However, myocytes are shown to undergo apoptosis under various pathological conditions of the heart [3–5]. Increase in sympathetic nerve activity in the heart is a central feature in patients with heart failure. Initially, an acute increase in sympathetic nerve activity helps maintain cardiac function. However, chronic increase in sympathetic nerve activity associates with adverse effects [3]. Stimulation of β-adrenergic receptors (β-AR) using norepinephrine and isoproterenol is shown to induce myocyte apoptosis *in vitro* and *in vivo* [4–7]. Significant advances have been made in understanding the signaling pathway leading to β-AR-stimulated myocyte apoptosis. Specific stimulation of β_1_-AR-Gs pathway induces apoptosis via the cyclic adenosine monophosphate / protein kinase A (cAMP/PKA)-dependent mechanism, while stimulation β_2_-AR-Gi pathway plays an anti-apoptotic role [8]. β-AR-stimulated myocyte apoptosis occurs via the activation of signaling kinases such as c-Jun N-terminal Kinases (JNKs), glycogen synthase kinase-3β (GSK-3β) and Ca^2+^/calmodulin kinase II [9–11], and involvement of endoplasmic reticulum stress and mitochondrial death pathways [12,13].

The Hippo-signaling pathway (also known as Salvador/warts/hippo pathway) plays a crucial role in controlling organ size by regulating cell proliferation and apoptosis. Serine/threonine kinases such as Warts (Wats), Hpo (MST1/2), Wts (Lats1/2) are the major components of the Hippo-signaling pathway [14,15]. Yes activated protein (YAP), a potent oncogene that is amplified in a variety of cancers, is a downstream effector in the Hippo pathway. Active YAP initiates the transcription of genes involved in cell proliferation. Mammalian sterile like kinase-1/2 (MST1/2) is the main kinase responsible for YAP phosphorylation [16]. Neurofibromin 2 (NF2; tumor suppressor protein) is suggested to act as a gatekeeper for the initiation of the Hippo-signaling pathway [17]. Evidence has been provided that NF2 acts as a bridge between cytoskeleton and membranous proteins, and thereby, modulating cell cycle progression and/or apoptosis [18]. NF2 undergoes post-translational modifications such as phosphorylation and sumoylation. Although NF2 can be sumoylated at various sites, lysine-76 is suggested to be a predominant sumoylation site [19]. Phosphorylation at serine-518 inactivates NF2, while promoting NF2 sumoylation [20]. NF2 phosphorylation at serine-518 and subsequent sumoylation modulate intermolecular and intramolecular interaction required for tumor suppressor activity as well as subcellular cytoplasmic/nuclear localization [19,21]. PKA (a cAMP-dependent kinase) and PAK (P21 activated kinase; a downstream target of Rac1/Cdc42) directly phosphorylate NF2 at serine-518 [21–23].

Within the heart, the Hippo-signaling pathway is suggested to play an important role in myocyte renewal and cardiac repair following myocardial infarction (MI). Inactivation of Salvador and Lats1/2 in the unstressed postnatal heart led to re-entry of myocytes into cell cycle and cytokinesis. Following MI, the Salvador and Lats1/2 deficiency associated with functional recovery of the heart and myocytes regeneration [24]. Conditional deletion of YAP from embryonic heart resulted in myocardial hypoplasia and embryonic lethality [25]. Cardiac myocyte-specific deletion of YAP in neonatal heart associated with progressive dilated cardiomyopathy and lethality between the age of 11 and 20 weeks [26]. On the other hand, cardiac myocyte-specific expression of constitutively activated YAP in the adult heart improved cardiac function, reduced scar formation and increased myocyte number post-MI [26]. Increased NF2 expression led to activation of MST1 and inhibition of YAP in mouse heart. Cardiac myocyte-specific NF2 knockout mice exhibited increased YAP expression and improved functional recovery following myocardial ischemia/reperfusion injury [27]. The involvement of NF2 and Hippo-signaling pathway as it relates to the activation of MST1/2 and YAP in β-AR-stimulated cardiac myocyte apoptosis remains to be investigated.

Here, we tested the hypothesis that β-AR stimulation affects post-translational modifications of NF2, and plays a pro-apoptotic role in β-AR stimulated myocyte apoptosis. The data presented here suggest that β-AR stimulation modulates NF2 phosphorylation and sumoylation via the involvement of β_1_-AR/PKA/cAMP pathway. NF2 plays a pro-apoptotic role in β-AR-stimulated myocyte apoptosis via the phosphorylation and inactivation of YAP, and involvement of mitochondrial death pathway.

## Materials and methods

### Experimental animals

The study used adult male Sprague-Dawley rats. The investigation conforms to the Guide for the Care and Use of Laboratory animals published by the US National Institutes of Health (NIH Publication No. 85–23, Revised 1996). All the experiments were performed in accordance with the protocols approved by the East Tennessee State University Committee on Animal Care.

### Cell isolation, culture and treatments

Calcium-tolerant ARVMs were isolated from the myocardium of adult male Sprague-Dawley rats (150–200 g) as described [28]. ARVMs, cultured for 24 h, were treated with isoproterenol (ISO; 10 μM; Sigma) or forskolin (FSK, 10 μM; Sigma) for 15 min. All the treatment dishes were supplemented with ascorbic acid (100 μM). CGP20712A (0.3 μM; Sigma), ICI 118551 (0.1 μM; Sigma) and H89 (20 μM, Calbiochem) were added for 30 min prior to ISO treatment. The inhibitors were maintained in the medium during the treatment period with ISO.

### H9C2 cell culture

The H9C2 rat cardiomyoblasts were obtained from American Type Culture Collection (Rockville, MD, USA) and maintained in DMEM supplemented with 10% fetal bovine serum and antibiotics. The cells were plated in 60 mm dishes with 150,000 cells per dish.

### Adenovirus infection

Adenoviruses expressing WT-NF2 (courtesy of Dr. Joseph Testa, Human Genetics Program, Fox Chase Cancer Center, Philadelphia, Pennsylvania) were propagated using HEK-293 cells. ARVMs were infected with the adenoviruses expressing WT-NF2 or green fluorescent protein (GFP) at a multiplicity of infection of 50-100/cell for 48 h.

### siRNA knockdown

Small interfering RNA (siRNA)-mediated knockdown of endogenous NF2 was performed in both ARVMs and H9C2 cells. For this, cells plated on coverslips or P60 mm dishes were washed with siRNA transfection medium (Santa Cruz Biotech) and incubated with 1 mL of transfection medium containing a pool of target specific NF2-siRNA (0.8μM; Santa Cruz Biotech) or negative control sequence siRNA (neg-siRNA; 0.8μM; Santa Cruz Biotech) at 37°C. Following 5 h of incubation, 1 mL of serum free DMEM supplemented with antibiotics was added to the dishes. The cells were then incubated for 24 or 48 h followed by treatment with ISO for 15 min or 24 h. The cells plated on coverslips were used for TUNEL assay, while cells plated on dishes were used for western blot analysis.

### Apoptosis

To detect apoptosis, ARVMs were plated on glass coverslips and stained using In Situ Cell Death Detection Kit (Roche Biochemicals) [29]. The percentage of TUNEL-positive cells (relative to total ARVMs) was determined by counting ∼200 cells in 10 randomly chosen fields per coverslip for each experiment.

### Cell fractionation

To prepare cytosolic and nuclear fractions, cells were lysed using cytoplasm lysis buffer (10 mM HEPES, pH 7.9), 10 mM KCl, 3 mM CaCl_2_, 1.5 mM MgCl_2_, 0.34 M sucrose, 1 mM DTT, 10% glycerol, 0.1% Triton X-100, protease and phosphatase inhibitors). For this, ten volumes of cell lysis buffer was added to one volume of packed cells. After resuspension and incubation on ice for 10 min, the cytosolic fraction was separated from the nuclear fraction by centrifugation at 500 × g for 7 min at 4°C. Collected nuclear pellet was resuspended in the nuclear lysis buffer (50 mM Tris-HCl,pH 7.9, 140 mM NaCl, 3 mM CaCl_2_, 1 mM EDTA, 1 mM DTT, 1% NP-40, 10% glycerol, protease and phosphatase inhibitors). The nuclear fractions were collected by centrifugation at 20,000 × g for 15 min at 4°C [30].

### Western blot analysis

Total cell lysates were prepared in lysis buffer (10 mM Tris-HCl; pH 7.4, 150 mM NaCl, 1 mM EGTA, 1 mM EDTA, 0.2 mM sodium orthovanadate, 0.5% Nonidet P-40, 1% Triton X-100, 1 mM PMSF, 8 μg/ml aprotinin and 2 μg/ ml leupeptin). Proteins from total cell lysates (75 μg) and cytosolic (10 μg) or nuclear fractions (100 μg) were analyzed by western blots as described [28]. The primary antibodies used were–NF2 (Santa Cruz), p-NF2 (serine-518; Cell Signaling), p-YAP (serine-397; Cell Signaling) and p-MST1/2 (Threonine183/180; Cell Signaling), Bax (Santa Cruz), Bcl_2_ (Santa Cruz) and p-JNKs (Threonine183/Tyrosine185, Threonine221/Tyrosine223, Millipore). Protein loading differences were normalized using GAPDH (Santa Cruz) or non-phospho-specific antibodies. Purity of nuclear and cytosolic fractions was assessed using poly (ADP ribose) polymerase (PARP; Cell Signaling) and GAPDH antibodies, respectively. Protein signals were visualized using ImageQuant LAS 500 imager and band intensities were quantified using ImageQuant TL1D v8 (GE Healthcare Life Sciences).

### Analysis of cytosolic cytochrome c

The cytosolic fractions were prepared and analyzed by western blots using anti-cytochrome c antibodies (Santa Cruz) as described [29].

### NF2 sumoylation assay

Total cell lysates (600 μg proteins) were incubated with 2 μg of anti-NF2 (Santa Cruz) antibodies overnight at 4°C. Protein A/G beads (60 μl, Thermo Scientific, Rockford, IL) were then added to the mixture and incubated for an additional hour. After incubation, the beads were washed six times using PBS. The proteins were eluted from the beads using 2X sample reducing buffer. The samples were separated by 7.5% SDS-PAGE and transferred to PVDF membranes. The membranes were then probed with anti-sumoylation antibodies (anti-SUMO-1, Cell Signaling).

### Statistical analyses

All data are expressed as mean ± SE. Statistical analysis was performed using Student’s t-test and one-way ANOVA followed by the Student-Newman-Keuls test. Probability (p) values <0.05 were considered to be significant.

## Results

### β-AR stimulation induces post-translational modifications of NF2

**Fig 1:**
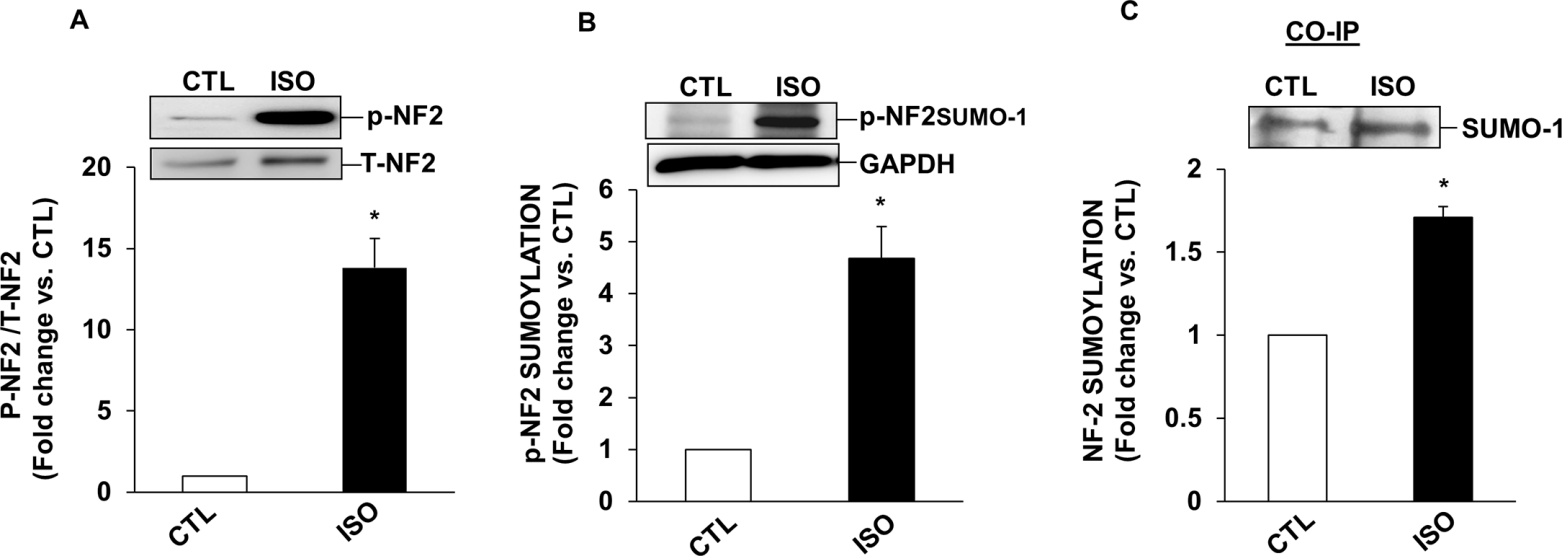
β-AR stimulation induces post-translational modifications of NF2 in ARVMs. ARVMs were treated with ISO (10 μM) for 15 mins. Total cell lysates were analyzed by western blot using phospho-specific anti-NF2 antibodies. This antibody recognized ~70 kDa (phosphorylated NF2; A) and ~100 kDa (phosphorylated and sumoylated NF2; B) proteins. The lower panels exhibit the mean data normalized to total NF-2 (T-NF2) or GAPDH, *p<0.05 vs CTL; n = 3–7. (C) To confirm NF2 sumoylation, cell lysates were immunoprecipitated with anti-NF2 antibodies. Immunoprecipitates were analyzed by western blot using anti-SUMO-1 antibodies. Panel C exhibits quantitative increase in NF2 sumoylation in response to ISO. *p<0.05 vs CTL; n = 3.

**Fig 2:**
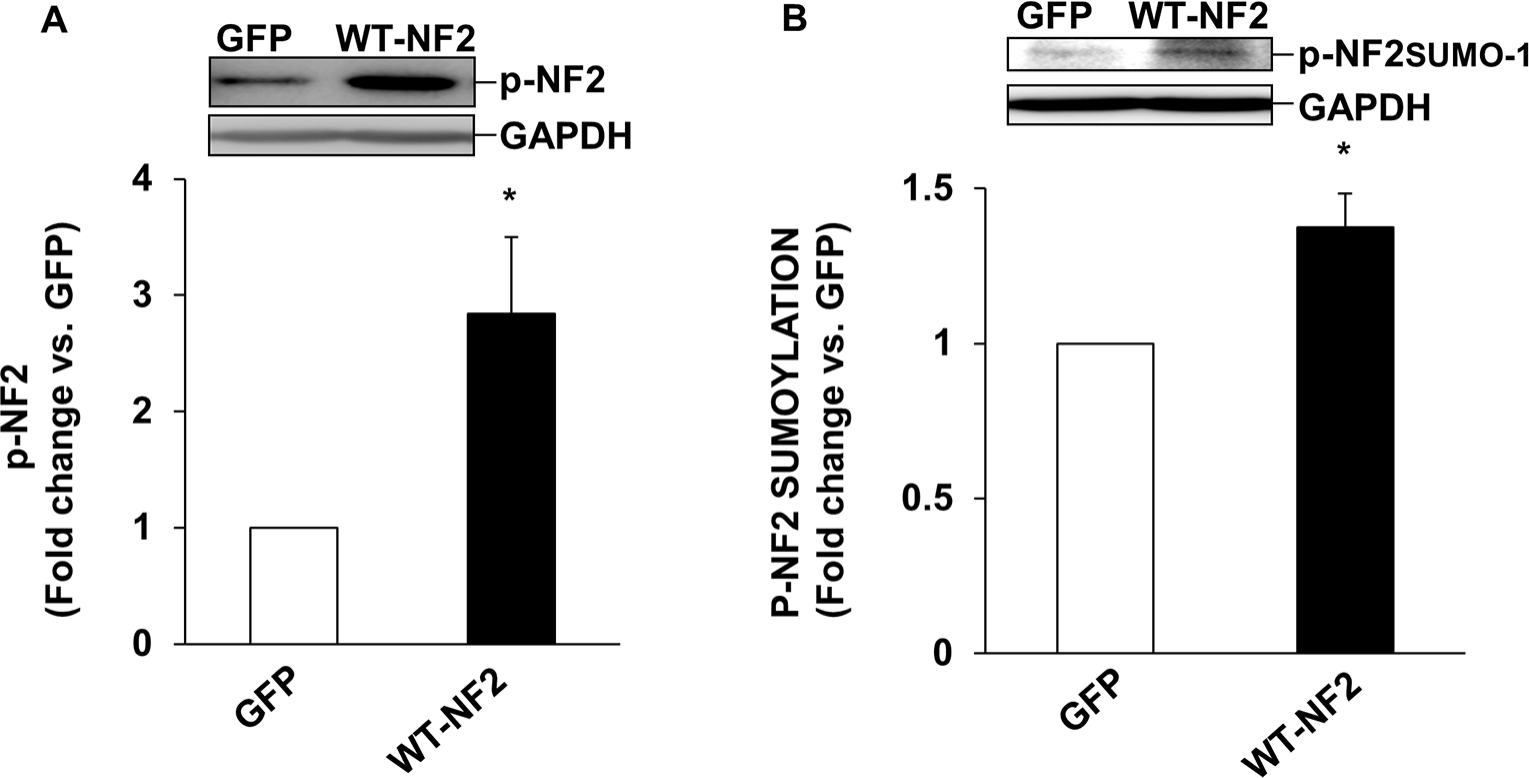
Adenoviral mediated expression of WT-NF2 induces post-translational modifications of NF2. ARVMs were infected with adenoviruses expressing WT-NF2 or GFP for 48 h. Cell lysates were analyzed by western blot using phospho-specific anti-NF2 antibodies. (A) Phosphorylated (~70 kDa) NF2; (B) Phosphorylated and sumoylated (~100 kDa) NF2. The lower panels exhibit the mean data normalized to GAPDH, *p<0.05 vs CTL; n = 6–7.

Post-translational modifications such as phosphorylation and sumoylation affect NF2 activity [20]. Phosphorylated NF2 protein exhibits an apparent molecular weight of ~70 kDa, while phosphorylated and sumoylated NF2 exhibits an apparent molecular weight of ~100kDa on SDS-PAGE. To investigate if β-AR stimulation affects NF2 phosphorylation and sumoylation, ARVMs were treated with ISO (10 μM, β-AR agonist) for 15 min. Analysis of total cell lysates using phospho-specific NF2 antibodies showed that ISO treatment significantly increases the band intensities of both ~70 kDa and ~100 kDa NF2 proteins, indicating that β-AR stimulation affects phosphorylation and sumoylation of NF2 (Fig 1A and 1B). To confirm the identity of ~100 kDa protein as NF2, co-immunoprecipitation assay was performed. For this, cell lysates were immunoprecipiated using anti-NF2 antibodies. Immunoprecipitates were then analyzed by western using anti-SUMO-1 antibodies. Anti-SUMO-1 antibodies recognized a band of ~100 kDa for NF2 suggesting that β-AR stimulation enhances NF2 sumoylation (Fig 1C). Adenoviral-mediated expression of wild-type-NF2 (WT-NF2) led to increased phosphorylation and sumoylation of NF2 when compared to cells expressing GFP (Fig 2A and 2B).

### Subcellular translocation of NF2 following β-AR stimulation

**Fig 3:**
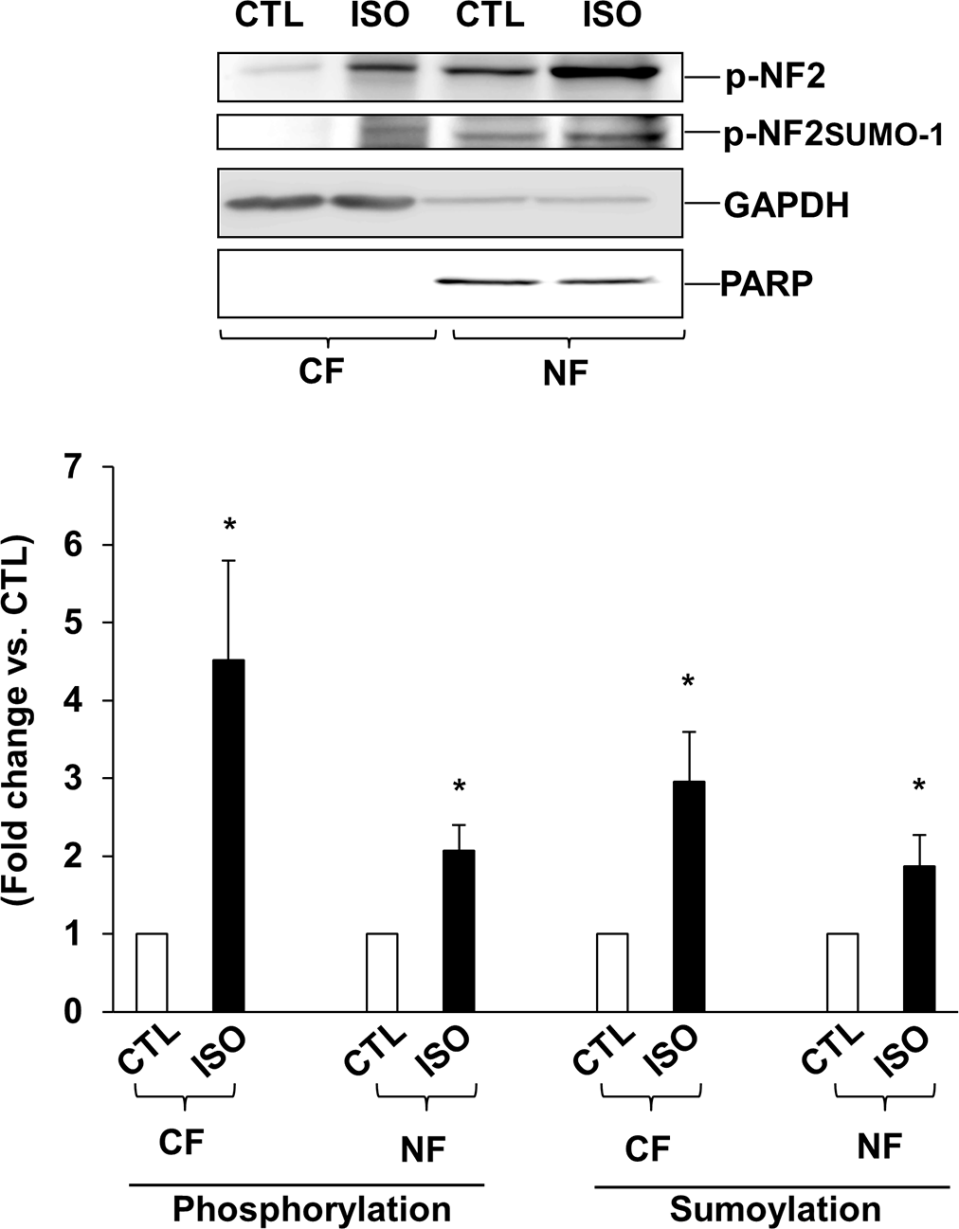
β-AR stimulation increases post-translational modifications of NF2 in nuclear and cytosolic fractions. ARVMs were treated with ISO for 15 mins. The cytosolic (CF) and nuclear fractions (NF) were analyzed by western blot using phospho-specific anti-NF2 antibodies. The lower panels exhibit the mean data normalized to GAPDH (CF) or PARP (NF), *p<0.05 vs CTL; n = 5–7.

In neonatal cardiac myocytes, NF2 is shown to localize in different cellular compartments including cytosol and nucleus [27]. To investigate if β-AR stimulation affects subcellular localization of NF2, cytosolic and nuclear fractions were analyzed by western blots using phospho-specific NF2 antibodies. This analysis showed a significant increase in phosphorylated (~70 kDa), and phosphorylated and sumoylated NF2 (~100 kDa) in both cytosolic and nuclear fractions when compared to their respective controls (Fig 3).

### Involvement of β-AR subtypes, adenylyl cyclase and PKA in post-translational modifications of NF2

**Fig 4:**
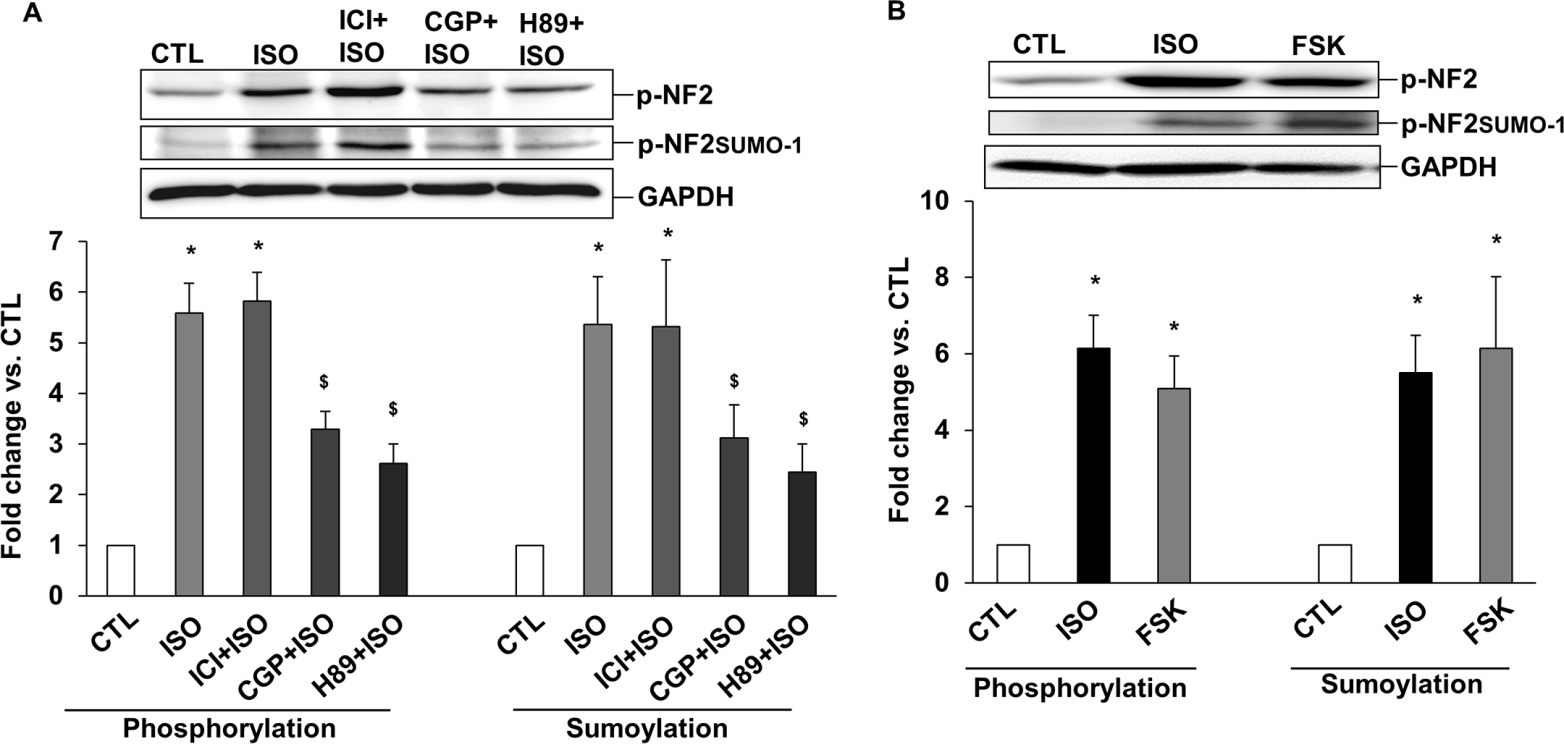
Involvement of β-AR subtypes, adenylyl cyclase and PKA in post-translational modifications of NF2. (A) ARVMs in CGP+ISO, ICI+ISO and H89+ISO groups were pretreated with CGP, ICI and H89, respectively, for 30 min prior to ISO treatment for 15 min. (B) ARVMs were treated with forskolin (FSK), an adenylyl cyclase activator, for 15 min. Cell lysates were analyzed by western blot using phospho-specific anti-NF2 antibodies. The lower panels exhibit the mean data normalized to GAPDH. *p<0.05 vs. CTL; $p<0.05 vs. ISO; n = 6–9.

Stimulation of β_1_-AR-PKA pathway induces apoptosis in ARVMs, whereas stimulation of β_2_-AR-Gi pathway inhibits apoptosis [31,32]. To investigate the involvement of β_1_-AR, β_2_-AR and PKA in phosphorylation and sumoylation of NF2, ARVMs were pretreated with CGP 20712A (0.3 μM; β_1_-AR-selective antagonist), ICI 118,551 (0.1 μM; β_2_-AR-selective antagonist) or H89 (20 μM; PKA inhibitor) for 30 min followed by treatment with ISO for 15 min. Western blot analyses of total cell lysates using phospho-specific NF2 antibodies showed that pretreatment with CGP and H89, not ICI, prevents ISO-mediated increase in the phosphorylation and sumoylation of NF2 (n = 6–9; Fig 4A). Direct activation of adenylyl cyclase using FSK (10 μM) also increased phosphorylation and sumoylation of NF2 (Fig 4B).

### NF2 and Hippo-signaling pathway

**Fig 5:**
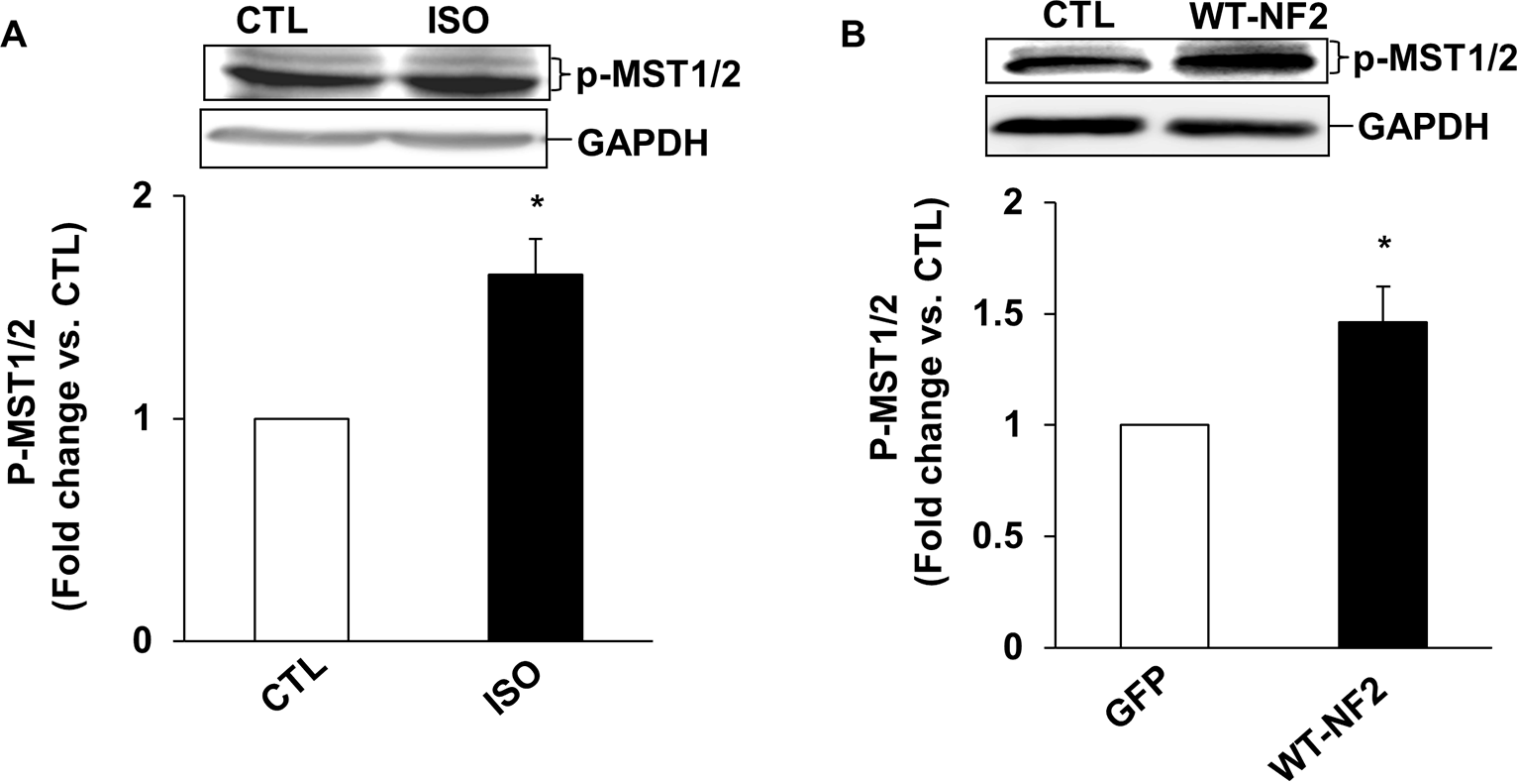
β-AR stimulation and adenoviral-mediated expression of NF2 increase phosphorylation of MST1/2 in ARVMs. ARVMs were treated with ISO for 15 min (A) or infected with adenoviruses expressing WT-NF2 or GFP for 48 h (B). Total cell lysates were analyzed by western blot using phosho-specific anti-MST1/2 antibodies. The lower panels exhibit the mean data normalized to GAPDH, *p<0.05 vs CTL or GFP; n = 5.

To investigate if NF2 acts upstream in the activation of Hippo signaling pathway, we first analyzed phosphorylation of MST1/2. For this, ARVMs were treated with ISO for 15 min. Analysis of total cell lysates using phospho-specific MST1/2 antibodies showed a significant increase in phosphorylation of MST1/2 in ISO-treated samples (n = 5; Fig 5A). Adenoviral-mediated expression of WT-NF2 also led to increased phosphorylation of MST1/2 (n = 5; Fig 5B).

**Fig 6:**
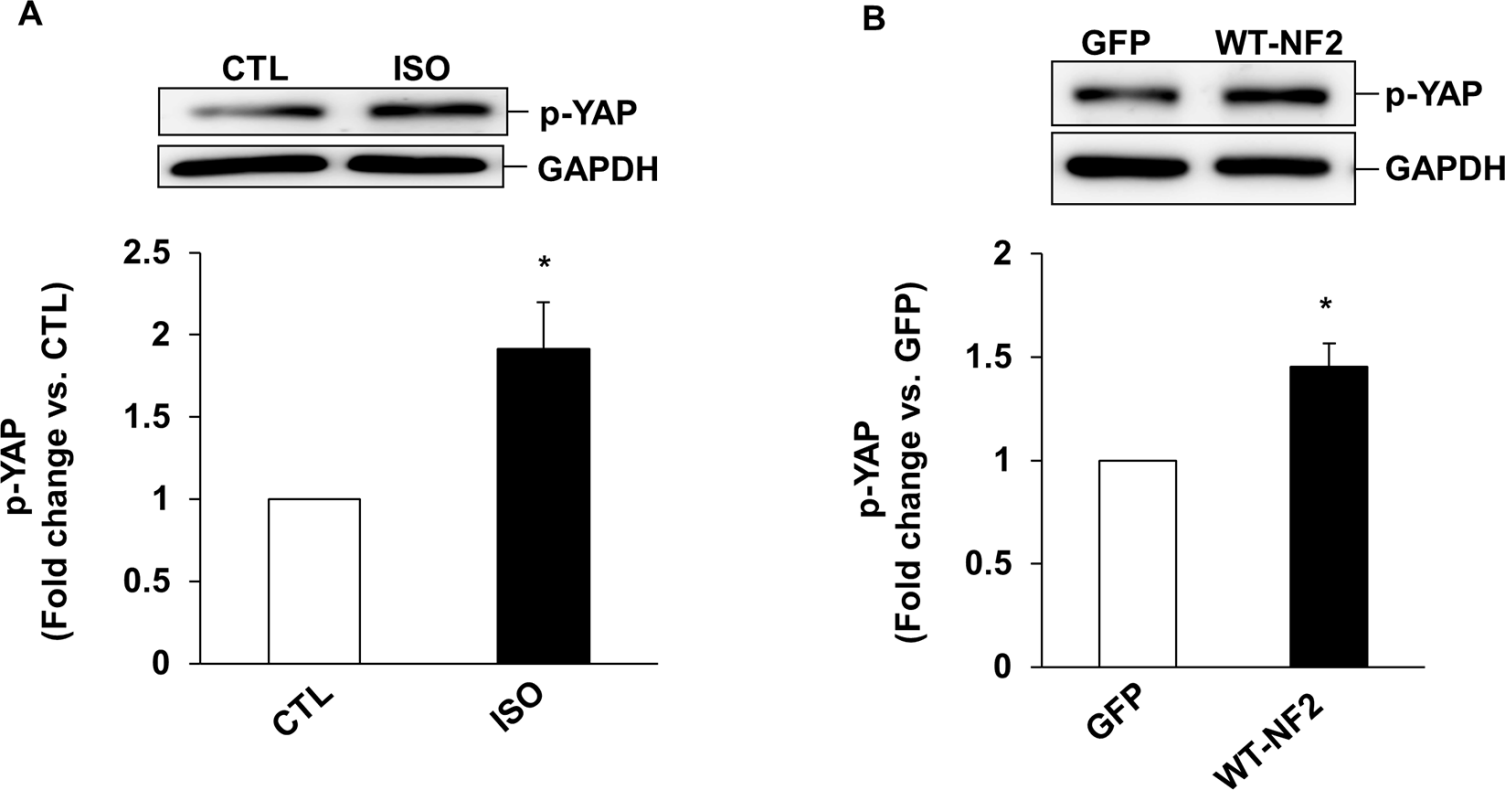
Stimulation of β-AR and adenoviral-mediated expression of NF2 increase YAP phosphorylation. ARVMs were treated with ISO for 15 min (A) or infected with adenoviruses expressing WT-NF2 or GFP for 48 h (B). Total cell lysates were analyzed by western blot using phospho-specific anti-YAP antibodies. There was no significant change in total YAP expression following ISO treatment. Therefore, data was normalized to GAPDH. The lower panels exhibit the mean data normalized to GAPDH, *p<0.05 vs CTL or GFP; n = 3–7.

YAP plays a vital role in regulating cell fate via various transcriptional mechanisms. Phosphorylation of YAP on serine-397 inactivates YAP, thereby decreasing its transcriptional activity [33,34]. To investigate if β-AR stimulation affects YAP phosphorylation, ARVMs were treated with ISO for 15 min. Western blot analyses of cell lysates using phospho-specific YAP antibodies showed a significant increase in phosphorylation of YAP (serine-397) in response to ISO (Fig 6A). Adenoviral-mediated expression of WT-NF2 also led to increased phosphorylation of YAP (Fig 6B).

**Fig 7:**
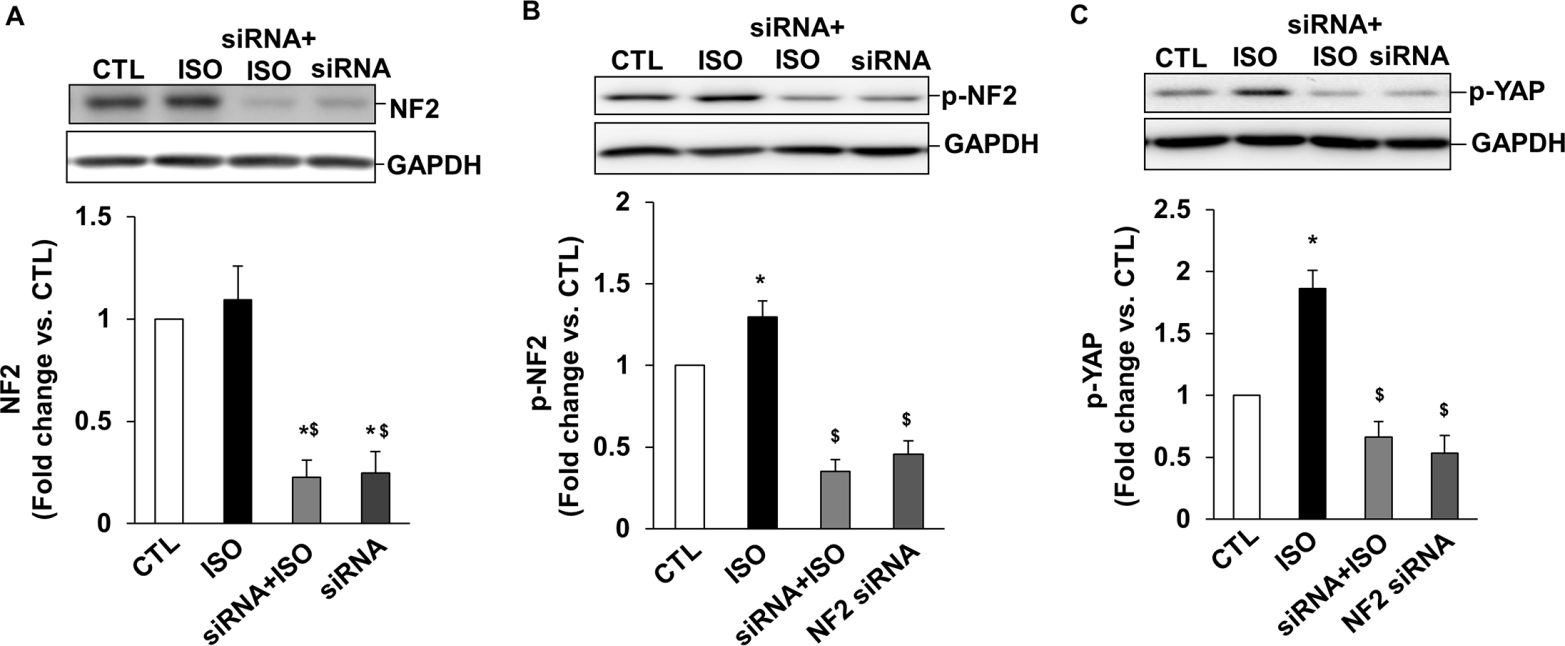
Knockdown of NF2 using siRNA decreases β-AR-stimulated increase in NF2 and YAP phosphorylation. H9C2 cells were transfected with NF2 siRNA for 48 h followed by treatment with ISO for 15 min. Cell lysates were analyzed by western blot using anti-NF2 (A), phospho-specific anti-NF2 (B) and phospho-specific anti-YAP (C) antibodies. The lower panels exhibit the mean data normalized to GAPDH, *P<0.05 vs.CTL; ^$^p<0.05 vs. ISO; n = 3.

To confirm if NF2 indeed acts upstream in phosphorylation of YAP, H9C2 cells were transfected with NF2 siRNA for 48 h followed by treatment with ISO for 15 min. Fig 7A shows siRNA-mediated knockdown of NF2. siRNA transfection knocked down NF2 protein levels by ~75% (Fig 7A). This knockdown of NF2 led to a significant decrease in NF2 and YAP phosphorylation in response to ISO (Fig 7B and 7C).

### NF2 and myocyte apoptosis

**Fig 8:**
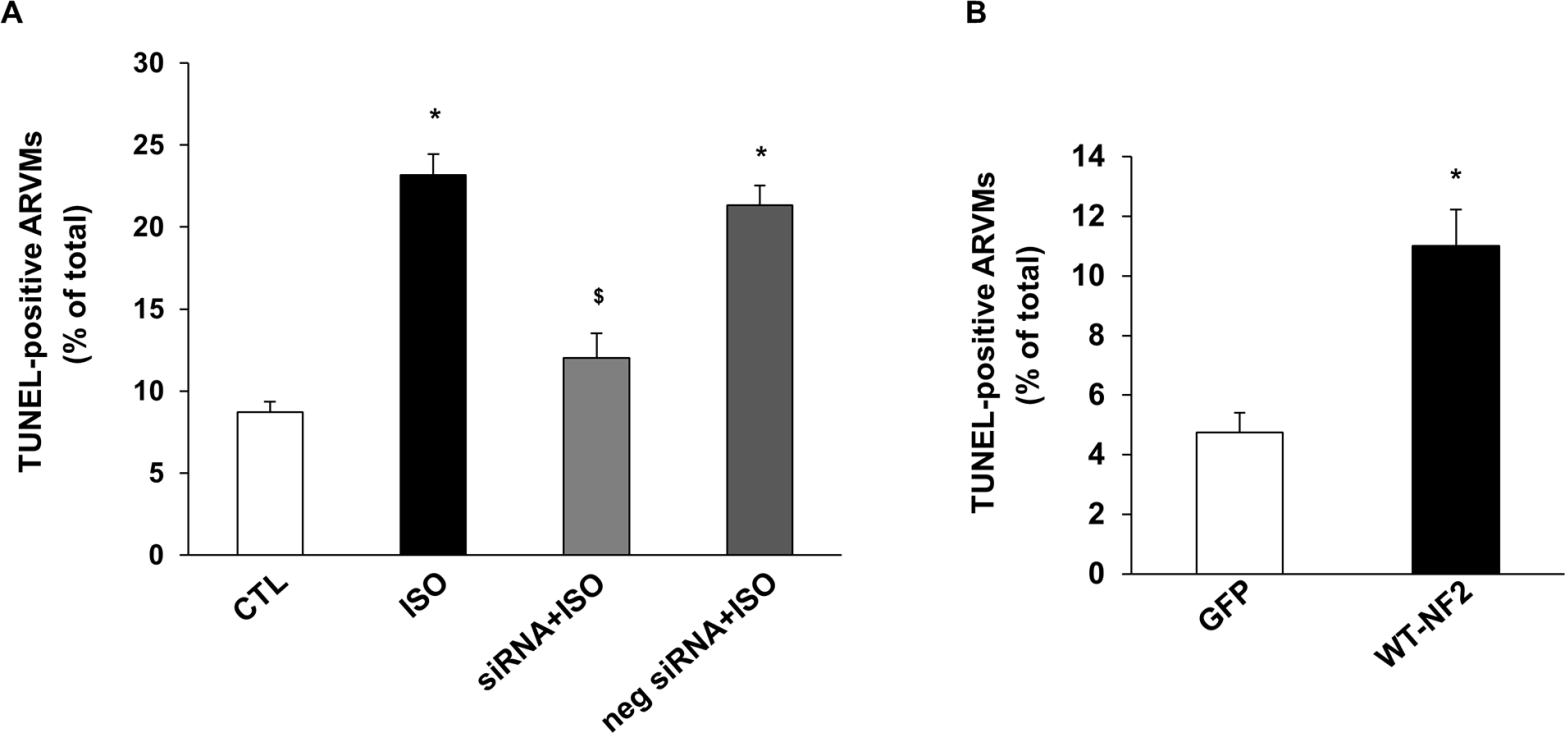
Knockdown of NF2 inhibits β-AR-stimulated apoptosis, while adenoviral-mediated expression of WT-NF2 induces apoptosis. (A) ARVMs were transfected with NF2 siRNA or negative control siRNA (neg siRNA) for 24 h followed by treatment with ISO for 24 h. Apoptosis was measured using TUNEL assay. *p<0.05 vs CTL; ^$^p<0.05 vs ISO or neg siRNA+ISO; n = 3–4. (B) ARVMs were infected with adenoviruses expressing WT-NF2 or GFP for 48 h. Apoptosis was measured using TUNEL assay. *p<0.05 vs GFP; n = 3–4.

To investigate the involvement of NF2 in β-AR-stimulated apoptosis, ARVMs were transfected with NF2 siRNA or neg siRNA for 24 h followed by treatment with ISO for 24 h. Analysis of apoptosis using TUNEL-assay showed that knockdown of NF2 significantly inhibits β-AR-stimulated apoptosis (CTL, 8.7±0.6; ISO, 23.15±1.3*; NF2 siRNA+ ISO, 11.99±1.5^$#^; negative siRNA+ISO, 21.33±1.2*; *p<0.05 vs CTL; ^$^p<0.05 vs ISO; ^#^p<0.05 vs ISO+neg siRNA; n = 3; Fig 8A). Transfection with negative siRNA had no effect in β-AR-stimulated increase in apoptosis. Adenoviral-mediated expression of WT-NF2 also significantly increased the number of apoptotic ARVMs (GFP, 4.74±0.67; WT-NF2, 11.00±1.21*; *p<0.05 vs GFP; n = 5; Fig 8B).

### NF2 expression activates mitochondrial death pathway

**Fig 9:**
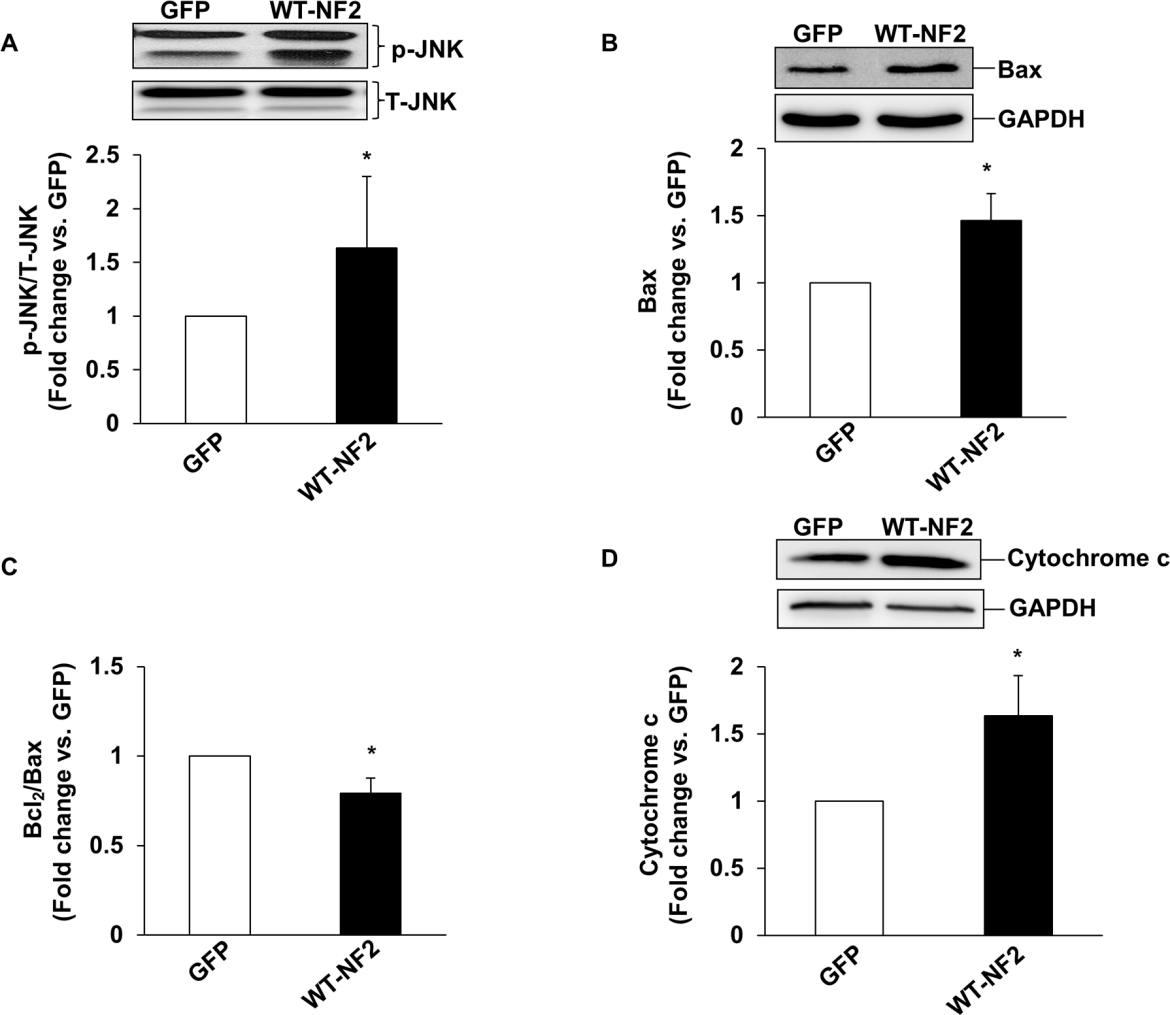
Adenoviral-mediated expression of NF2 activates mitochondrial death pathway in ARVMs. ARVMs were infected with adenoviruses expressing WT-NF2 or GFP for 48 h. Cell lysates were analyzed by western blots using phospho-specific anti-JNKs (A), anti-Bax (B), and anti-Bcl_2_ antibodies (data not shown), while cytosolic fractions were analyzed by western blot using anti-cytochrome c (D) antibodies. The lower panels exhibit the mean data normalized to total JNKs (T-JNKs) or GAPDH. Fig 9 (C) represents Bcl_2_/Bax ratio. *p<0.05 vs GFP; n = 4–6.

In ARVMs, β-AR-stimulated apoptosis occurs via the involvement of JNKs and mitochondrial death pathway [9]. To investigate the involvement of mitochondrial death pathway, we measured activation of JNKs, expression of Bax and Bcl_2_ and levels of cytosolic cytochrome c in ARVMs infected with adenoviruses expressing WT-NF2 for 48 h. Western blot analyses of cell lysates using phospho-specific JNKs antibodies showed a significant increase (~1.6-fold) in JNKs (46 and 54 KDa) phosphorylation versus cells expressing GFP (Fig 9A). NF2 expression also increased protein levels of Bax, a pro-apoptotic protein, by ~1.5-fold (Fig 9B). Protein levels of Bcl_2_ remained unchanged (data not shown). Bcl_2_/Bax ratio was significantly lower in cells expressing NF2 (Fig 9C). Levels of cytosolic cytochrome c were significantly higher (~1.6-fold; Fig 9D) in cells expressing WT-NF2 when compared to cells expressing GFP.

## Discussion

Increased sympathetic nerve activity associates with increased myocyte apoptosis *in vitro* and in mouse models of heart disease [7,8,35–37]. Previously, we provided evidence that β-AR stimulated cardiac myocyte apoptosis occurs via the involvement of endoplasmic reticulum stress and mitochondrial death pathways [9,13]. This is the first study investigating the post-translational modifications of NF2 in response to β-AR stimulation in adult cardiac myocytes, and its role in myocyte apoptosis. The major findings of the present study are—1) β-AR stimulation and adenoviral-mediated expression of NF2 induces post-translational modifications (phosphorylation and sumoylation) of NF2; 2) β-AR stimulation increases nuclear translocation of NF2; 3) NF2 phosphorylation and sumoylation occur via the involvement of β_1_-AR/PKA/cAMP pathway; 4) β-AR stimulation and adenoviral-mediated expression of WT-NF2 increases phosphorylation of MST1/2 and YAP; 5) knockdown of NF2 using siRNA inhibits β-AR stimulated phosphorylation of NF2 and YAP in H9C2 cells; 6) knockdown of NF2 using siRNA inhibits β-AR stimulated apoptosis, while expression of WT-NF2 induces apoptosis in ARVMs; and 7) expression of WT-NF2 associates with activation of the mitochondrial death pathway of apoptosis.

During the normal developmental process, cell growth is regulated by controlled cell proliferation and apoptosis. NF2, a gatekeeper of the Hippo-signaling pathway, plays an important role in cell cycle arrest and apoptosis [38]. Mutations of NF2 are frequently observed in tumors of the nervous system. NF2 is essential in the early developmental phase of embryogenesis in mice as mutations in this stage are generally lethal [39]. Post-translational modifications such as sumoylation, ubiquitylation, acetylation, and methylation are important protein modifiers that determine the activation, deactivation or subcellular localization of signaling proteins [40]. Post-translational modifications of NF2 are essential for its conversion between the active and inactive forms. Kinases such as PKA and PAK are suggested to phosphorylate NF2 at serine-518 which in turn promotes NF2 sumoylation [20]. In a majority of cell lines, endogenous NF2 molecular weight of ~70 kDa is shown to be available for sumoylation with the band appearing at ~100 kDa on a SDS-PAGE[19]. Here, we provide the evidence that β-AR stimulation induces post-translational modifications of NF2 in ARVMs. Phospho-specific anti-NF2 antibodies recognized bands with apparent molecular weights of ~70 and ~100 kDa for NF2. Both phosphorylation and sumoylation were significantly higher in ISO-treated cells. Co-immunoprecipitation assay confirmed the identity of ~100 kDa protein as sumoylated NF2. In addition, adenoviral-mediated expression of WT-NF2 led to increased phosphorylation and sumoylation of NF2. Protein sumoylation generally associates with nuclear transport [41]. NF2 has a nuclear localization motif at the N-terminal domain that helps NF2 to shuttle between the cytosol and the nucleus [42]. Sumoylation increases nuclear localization of NF2 [19]. β-AR stimulation enhanced phosphorylation and sumoylation of NF2 in both cytosolic and nuclear fractions. The observed increases in phosphorylated as well as sumyolated NF2 suggest activation of NF2 in response to β-AR-stimulation. This observation is further supported by increased phosphorylation of downstream targets MST1/2 and YAP, in response to β-AR stimulation.

The activation of β_1_-AR-Gs pathway increases apoptosis, while activation of β_2_-AR-Gi pathway plays an anti-apoptotic role in β-AR-stimulated apoptosis in ARVMs [31,43,44]. β-AR stimulation results in activation of the downstream adenylyl cyclase-cAMP-PKA pathway [45]. PKA and PAK are the known kinases involved in phosphorylation of NF2 on serine-518 in various cell types [21,46,47]. Here, inhibition of β_1_-AR and PKA, but not β_2_-AR, inhibited β-AR-stimulated increase in post-translational modification of NF2, suggesting the involvement of β_1_-AR subtype and PKA in the phosphorylation and sumoylation of NF2. Activation of β-AR in cardiac myocytes increases the cellular concentration of cAMP [48]. Previously, direct stimulation of adenylyl cyclase using FSK is shown to induce apoptosis in ARVMs [49]. Similar to β-AR stimulation, FSK treatment for 15 min increased phosphorylation and sumoylation of NF2, suggesting the involvement of adenylyl cyclase and cAMP in this process. Together these data suggest involvement of β_1_-AR/PKA/cAMP pathway in post-translational modifications of NF2.

YAP, a transcriptional cofactor of the Hippo-signaling pathway, promotes myocyte proliferation in embryonic mouse heart by activating the insulin-like growth factor and Wnt signaling pathways. It also plays an essential role in early stages of embryonic heart development. NF2 serves as an activator of MST1/2, and MST1/2 is the main kinase responsible for phosphorylation and inactivation of YAP. Here, β-AR stimulation and expression of WT-NF2 increased YAP phosphorylation (inactivation) in both ARVMs and H9C2 cells, while siRNA mediated knockdown of NF2 inhibited β-AR-stimulated YAP phosphorylation. β-AR stimulation and expression of WT-NF2 also increased phosphorylation (activation) of MST1/2. These data suggest that NF2 acts upstream in the phosphorylation MST1/2 (activation) and YAP (inactivation) in response to β-AR stimulation.

Mitochondria play a vital role in cellular metabolism, energy production and determination of cell fate with respect to survival and apoptosis [50]. Cardiac mitochondrial dysfunction is suggested to associate with the progression of heart failure [51]. Translocation of Bax to the mitochondria and the subsequent extrusion of cytochrome c into the cytosolic environment are considered as major events in mitochondrial dysfunction [52]. In cardiac myocytes, β-AR-stimulated apoptosis occurs via the JNK-dependent activation of the mitochondrial death pathway [9]. The data presented here demonstrate that siRNA-mediated knockdown of NF2 inhibits β-AR-stimulated myocyte apoptosis, while expression of WT-NF2 activates JNKs, and increases Bax expression, cytosolic cytochrome c levels, and myocytes apoptosis. Together, these observations suggest that NF2 plays a pro-apoptotic role, and NF2 may act upstream in the activation of JNKs-dependent activation of mitochondrial death pathway.

**Fig 10:**
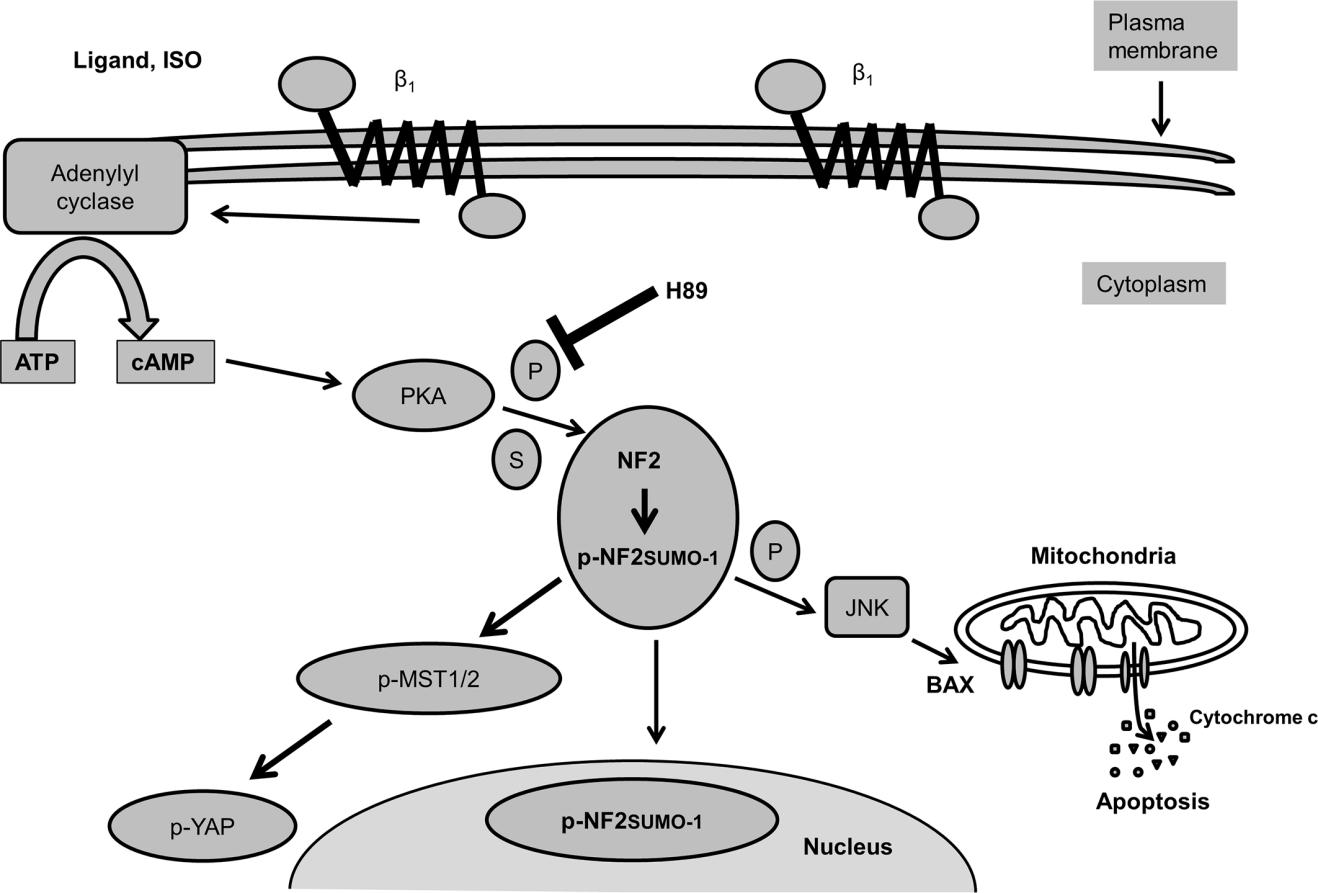
Proposed schematic representation of signaling pathway involved in β-AR-stimulated increase in post-translational modifications of NF2. β-AR-stimulation using isoproterenol increases phosphorylation and sumoylation of NF2. This pathway may involve β_1_-AR and cAMP/PKA pathway since β_1_-AR antagonism and H89 inhibit β-AR-stimulated increase in NF2 phosphorylation and sumoylation. Direct activation of adenylyl cyclase mimics the effects of β-AR-stimulation on NF2 phosphorylation and sumoylation. β-AR-stimulation increases levels of phosphorylated and sumoylated NF2 in the nucleus. β-AR-stimulation increases phosphorylation of MST1/2 and YAP, downstream targets of NF2. Expression of WT-NF2 using adenoviruses stimulates mitochondrial death pathway by activating JNKs, increasing Bax expression and enhancing cytosolic levels of cytochrome c.

Our observations support the proposed signaling cascade involved in β-AR-stimulated increase in post-translational modifications of NF2 and apoptosis (Fig 10). β-AR-stimulated modifications and activation of NF-2 and subsequent inactivation of YAP may play critical roles in myocyte apoptosis during sympathetic overstimulation.

## Conclusion

Deficiency of NF2 and YAP play a cardioprotective role against ischemia/reperfusion injury [27,53]. Our study provides evidence that β-AR stimulation induces post-translational modifications of NF2 via the involvement of the β_1_-AR/PKA/cAMP pathway. These post-translational modifications of NF2 may affect activation and localization of NF2, and activation of components of the Hippo-signaling pathway. The data also provide evidence that NF2 plays a pro-apoptotic role in β-AR-stimulated apoptosis via the involvement of mitochondrial death pathway in cardiac myocytes. A clear understanding of the signaling pathways involved in the regulation of expression and activity of components of Hippo-signaling pathway, and their role in cardiac myocyte apoptosis may uncover novel therapies for the treatment of heart failure.
